# Structure and Stability of Human Telomeric G-Quadruplex with Preclinical 9-Amino Acridines

**DOI:** 10.1371/journal.pone.0057701

**Published:** 2013-03-15

**Authors:** Ruben Ferreira, Roberto Artali, Adam Benoit, Raimundo Gargallo, Ramon Eritja, David M. Ferguson, Yuk Y. Sham, Stefania Mazzini

**Affiliations:** 1 Institute for Research in Biomedicine, IQAC-CSIC, CIBER-BBN Networking Center on Bioengineering, Biomaterials and Nanomedicine, Barcelona, Spain; 2 Scientia Advice di Roberto Artali, Lissone, Italy; 3 Department of Medicinal Chemistry, University of Minnesota, Minneapolis, United States of America; 4 Department of Analytical Chemistry, University of Barcelona, Barcelona, Spain; 5 Center for Drug Design, Academic Health Center, University of Minnesota, Minneapolis, United States of America; 6 Department of Food, Environmental and Nutritional Sciences (DEFENS), Department of Organic Chemistry, University of Milan, Milan, Italy; University of Quebect at Trois-Rivieres, Canada

## Abstract

G-quadruplexes are higher-order DNA structures formed from guanine-rich sequences, and have been identified as attractive anticancer drug targets. Elucidating the three-dimensional structure of G-quadruplex with 9-amino acridines and the specific interactions involved in binding selectivity are the key to understanding their mechanism of action. Fluorescence titration assays, competitive dialysis and NMR studies have been used to study the binding specificity of 9-amino acridines to DNA. Structural models of the complexes with the telomeric DNA G-quadruplex based on NMR measurements were developed and further examined by molecular dynamics simulations and free energy calculations. Selective binding of 9-amino acridines for G-quadruplex sequences were observed. These compounds bind between A and G-tetrads, involving significant π-π interactions and several strong hydrogen bonds. The specific interactions between different moieties of the 9-amino acridines to the DNA were examined and shown to play a significant role in governing the overall stabilities of DNA G-quadruplex complexes. Both 9-amino acridines, with similar binding affinities to the G-quadruplex, were shown to induce different level of structural stabilization through intercalation. This unique property of altering structural stability is likely a contributing factor for affecting telomerase function and, subsequently, the observed differences in the anticancer activities between the two 9-amino acridines.

## Introduction

In the last years, tricyclic acridine-containing compounds have been investigated as small molecule chemotherapeutic anticancer agents [1], [2]. Studies on the mechanism of action of acridine drugs have shown these compounds are potent inhibitors of topoisomerase and telomerase function in replicating cells [3], which ultimately leads to apoptosis and cell death.

Topoisomerase alters DNA topology through the decatenation and relaxation of the supercoiled chromosomal DNA [4]. By unwinding the double-stranded DNA, this essential enzyme enables normal cellular DNA replication and transcription [4]. DNA topoisomerases exist in various eukaryotic and prokaryotic forms [5] and are classified in two large groups, namely type I and type II. Anti-cancer drugs targeting topoisomerase can also be classified as either catalytic inhibitors or “topoisomerase poisons” depending on their mechanism of action [6]. The latter can be further sub-classified into two groups: non-intercalating compounds such as etoposide, and intercalators such amsacrine and doxorubicin [7].

The telomere is a highly repetitive DNA region located at the end of a linear chromosome. Its function is to protect the terminal ends of chromosomes from being recognized as damaged DNA and allows faithful chromosome replication during the cell cycle [8], [9]. Human telomeric DNA contains tandem repeats of the sequence 5′-TTAGGG-3′. This guanine-rich strand can fold into a four-strand G-quadruplex structure involving G-tetrads, which are currently an attractive target for the development of anti-cancer drugs [10], [11], [12].

A wide range of small molecules have been studied as G-quadruplex-binding and stabilizing ligands [13]. Most of these share common structural features, namely: (i) a planar heteroaromatic chromophore, which stacks by π-π interactions onto the G-quartet motif at the terminus of a G-quadruplex; and (ii) short alkyl chain substituents usually terminated by an amino group that is fully cationic at physiological pH. The precise nature of these substituents has been found to influence G-quadruplex affinity and selectivity [12], [14], [15].

Guanine rich sequences are not only present in telomers [8], [9] but also in transcriptional regulatory regions of important genes implicated in cancer such as oncogenic promoters of human vascular endothelial growth factor VEGF [16], *c-myc* [17], *c-kit* [18], *bcl*-2 [19], N-*ras* [20], K-*ras* [21], and RET [22]. There are strong evidences that the transcriptional control of these genes can be modulated by G-quadruplex interacting agents [23], [24]. For this reason ligands that selectively bind and stabilize G-quadruplex have become interesting anticancer drugs [25], [26].

Ferguson and coworkers have recently described a series of 9-aminoacridine compounds that inhibit topoisomerase II activity, these compounds were initially synthesized as part of a library screening study to identify anti-Herpes agents [27]. These compounds have been shown to be active against a variety of cancer cells in vitro and in vivo [14]. Mechanistic studies have shown these compounds bind DNA and block the formation of covalent DNA-Topo II complexes [28], stalling the cell cycle in the G1->S phase. This, in turn, induces apoptosis and programmed cell death. Previous studies, however, have shown that acridine compounds are also capable of binding telomeric G-quadruplex structures with high affinity [29], [30]. This is yet another route by which compounds of this type may disrupt DNA replication in rapidly dividing cancer cells. In this study, we examine the binding affinity of several 9-amino acridines to quadruplex DNA using competitive dialysis and spectroscopic techniques. High field 2D-NMR experiments are also performed to provide insight to the structural interactions that stabilize the drug-DNA complex. Finally, the NOE data is applied to generate structural models for evaluation using molecular mechanics and dynamics techniques.

## Results and Discussion

### Competitive Dialysis and Fluorescence Titration

In order to evaluate the selectivity of the compounds for DNA structures or sequences, a competitive dialysis experiment was performed using 11 oligonucleotides (Table 1) representing several nucleic acid structures [31], [32], [33]. We used T20 and the C-rich complementary strand of bcl-2 as model compounds for single stranded DNA sequences. As duplexes we used the self-complementary sequences *Dickerson-Drew* dodecamer and a 26 mer (ds26). A parallel and an antiparallel triplex were also selected. Finally several DNA sequences known to form G-quadruplex were selected: TG4T [34] is a tetramolecular parallel G-quadruplex, the TBA [35] is the antiparallel thrombin-binding aptamer, the HT24 [36] is the human telomerase sequence, the cmyc and bcl-2 [19] are promoter sequences of *c-myc* and *bcl-2* protooncogenes.

**Table 1:** Oligonucleotides sequences used in this study.

DNA code	5′-sequence-3′
**T20**	TTT TTT TTT TTT TTT TTT TT
**24bclc**	CCC GCC CCC TTC CTC CCG CGC CCG
**6-mer**	CGA TGC
**Dickerson**	CGC GAA TTC GCG
**ds26**	CAA TCGGAT CGA ATT CGA TCC GAT TG
**GA triplex**	GAA AGA GAGGAG GCC TTT TTG GAG GAG AAG+CCT CCT CTC TTT C
**TC triplex**	CCT CCT CTC TTT CCC TTT TTC TTT CTC TCC TCC+GAA AGA GAG GAG G
**TG_4_T**	TGG GGT
**TBA**	GGT TGG TGT GGT TGG
**HT24**	TAG GGT TAG GGT TAG GGT TAGGGT
**Htel**	TTA GGG
**HtelT**	TTA GGG T
**24bcl**	CGG GCG CGG GAGGAA GGG GGC GGG
**cmyc**	GGG GAG GGT GGG GAG GGT GGG GAA GGT GGG G
**ds6**	CGA TCG
**ds8**	GCG ATC GC
**ds24**	AAG AAT TCT TAA GAA TTC TTA ATT

**Figure 1:**
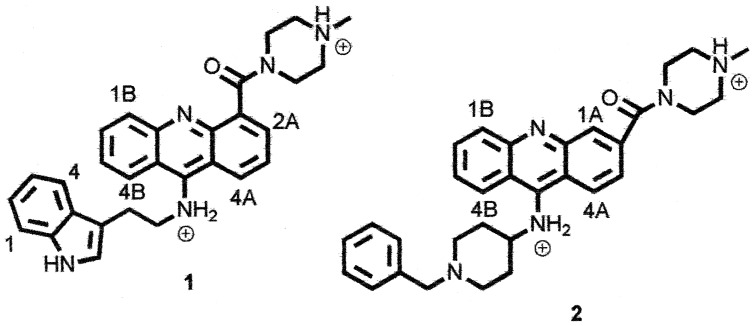
Structure of 1 and 2.

**Figure 2:**
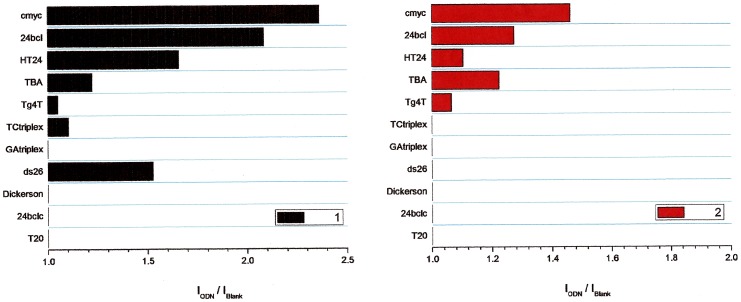
Results obtained by the competitive dialysis assays. The amount of ligand bound to each DNA structure is shown as a bar graph.

Competitive dialysis experiments show clear differences on the affinity of ligands to a different DNA structures (Figure 1 and Figure 2). Higher affinities are found in G-quadruplex sequences present on the promoter regions of *c-myc* and *bcl-2* oncogenes and the human telomere sequence. Ligand **2** has a clear selectivity for G-quadruplex-forming DNA sequences while compound **1** has also an affinity for duplex ds26 as show in Figure 2.

Finally, the stability of the interaction complex formed by ligand **1** and **2** with G-quadruplex Htel and duplex ds6 was quantified by recording the fluorescence spectra of a solution of the ligand after the addition of increasing amounts of oligonucleotide. Changes in the fluorescence spectra upon the addition of G-quadruplex were observed in **1** and **2** (Figure S1). No significant changes in the fluorescence spectra were observed upon the addition of duplex. The increase of the fluorescence emission of these compounds reflects their interaction with the G-quadruplex Htel.

Table S1 shows the logarithm of the binding constants calculated using Equispec program assuming a 1:1 stoichiometry DNA:ligand for the interaction complex. The calculated values are around 105 M-1 which, according to literature, could be related to intercalating species 37]. Groove-binding compounds are expected to show larger association constants 38].

The high affinity of 9-amino acridines **1** and **2** for G-quadruplex sequences and the strong interest in the bibliography for G-quadruplex binders [25], [26] prompted us to study in detail the complex formed by the 9-aminoacridines and G-rich sequences.

### NMR 9-amino Acridines-DNA Experiments

NMR studies were performed to confirm and elucidate the structure of the complexes formed between compounds **1** and **2** and DNA. The oligomers ds6, ds8, ds24, ds26 and Htel (Table 1) were used as models for double stranded DNA and G-quadruplex parallel structures respectively. ^31^P and ^1^H resonance experiments were performed to derive both the mode of binding and the details of the molecular model. Assignment of the phosphorous, of the exchangeable and non-exchangeable protons for all oligonucleotides was carried out on the basis of previously reported assignments [39], [40].

The sequential assignments in free and bound oligonucleotides were performed by applying well established procedures for the analysis of double stranded and G-quadruplex structures. ^1^H assignments for **1** and **2** in absence of DNA were performed by using ROESY, NOESY, TOCSY and COSY experiments. Complete assignments are reported in Table 2 and 3.

**Table 2:** ^1^H chemical shift assignments for 1 and 2 (δ) and shift variation of 1 (Δδ) in the presence of Htel^a^.

1	δ free	δ bound Htel	Δδ (*δ*bound–*δ*free)	2^b^	δ free
4A	8.36	8.10	−0.26	1B	8.31
1B	8.28	8.10	−0.18	3A	8.22
3B	7.92	7.74	−0.18	4B	7.92
4B	7.87	7.74	−0.13	1A	7.90
2A	7.80	7.66	−0.14	2B	7.80
2B	7.55	7.48	−0.07	3B	7.49
3A	7.55	7.20	−0.35	2A	7.30
H1	7.27	7.00	−0.27	H Phe	7.26–7.40
H4	7.25	7.00	−0.25	CH_2_ ring1	4.00, 3.75, 3.49, 2.60, 2.45
H2	7.10	6.61	−0.49	CH_2_ ring2	2.85, 2.15, 2.05, 1.86
H3	6.76	6.42	−0.34	CH_2_ benz	3.55
Hind	6.94	6.60	−0.34	CH	2.62
NH ind	9.75	9.60	−0.15	NCH_3_	2.27
CH_2_-NHind	3.45	n.d.	−	−	−
CH_2_-NH	4.60	n.d.	−	−	−
NCH_3_	2.20	n.d.	−	−	−
CH_2_ ring	2.8,2.93.4,3.9	2.27 2.82, 4.10	−0.53, −0.63 −0.58,0.20	−	−

a Measured in ppm at 25°C, *R* = [drug]/[DNA] = 3;

b Tentatively the chemical shift variations are within −0.08 ppm and −0.5 ppm.

**Table 3:** ^1^H chemical shift assignments for Htel (δ) in the presence of 1 and 2^a^.

TTAGGG/1	NH	H2/H8/H6	H1′	H2′,H2′′	H3′	H4′	CH_3_
**T1**	−	7.43	6.05	2.42, 2.11	4.86	4.11	1.86
**T2**	−	7.43	6.05	2.42, 2.18	4.86	4.24	1.86
**A3**	−	8.10,8.51	6.38	2.98, 2.98	5.19	4.57	−
**G4**	11.23	7.72	6.07	2.93, 2.60	5.04	4.32	−
**G5**	10.89	7.48	6.07	2.91, 2.68	5.03	4.39	−
**G6**	10.53	7.37	6.12	2.87, 2.66	4.88	4.53	−
**TTAGGG/2**	**NH**	**H2/H8/H6**	**H1′**	**H2′,H2′′**	**H3′**	**H4′**	**CH_3_**
**T1**	−	7.60	6.06	2.48,2.28	4.85	3.96	1.93
**T2**	−	7.46	6.23	2.49, 2.25	4.93	4.28	1.93
**A3**	−	8.10,8.54	6.42	3.02, 3.02	5.22	4.61	−
**G4**	11.35	7.88	6.11	3.02, 2.76	5.03	4.43	−
**G5**	10.96	7.59	6.20	3.02,2.83	5.17	4.46	−
**G6**	10.61	7.46	6.23	3.00, 2.77	4.96	4.62	−

a Measured in ppm at 25°C, *R* = [drug]/[DNA] = 3.0.

### NMR 9-amino Acridines and G-quadruplex Experiments

**Figure 3:**
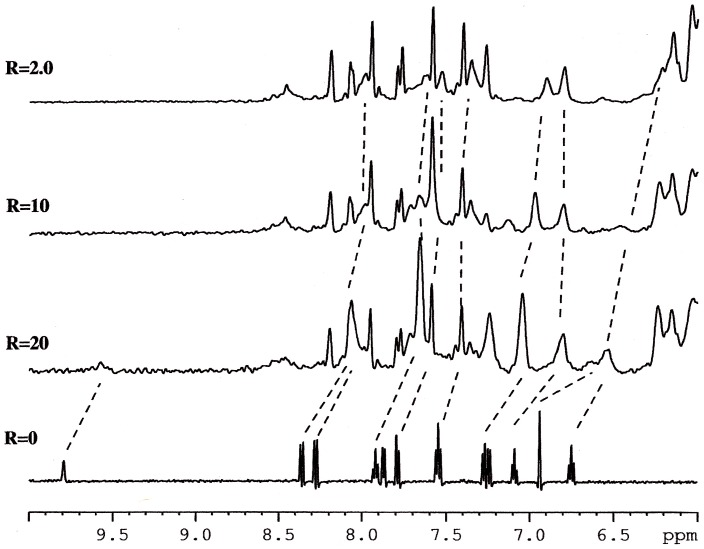
^1^H NMR spectra showing NH of indole moiety and aromatic protons of 1 in the free state (R = Htel/[1] = 0) and at different R. High and low R values must be related to the free and bound state of 1 in solution respectively.

Titration experiments performed with **1** and **2** on the solution of Htel show that the proton resonances of the drug become broad and move up-field with respect to the free drug, just after the addition of a small quantity, *i.e.* with *R* = [drug]/[DNA] = 0.25. Increasing the *R* value from 0.25 to 3, the shielding of the drug protons carries on and it spread over the whole drug molecule. In order to better identify the drug protons in the complex, the inverse titration experiment was performed, by adding increasing amounts of DNA, from R = 20 to 2.0, to a solution of **1** at constant concentration (0.2 mM). Figure 3 depicts the chemical shift variation observed for protons of **1** during the titration experiment. The chemical shift at higher R values must be related to the free drug in solution, the addition of the oligonucleotide induces a shielding of the drug protons. When an excess of oligonucleotide is reached (low values of R), the drug is found predominantly in a bound state. Actually, the chemical shift variation of a ligand is due to the sum of different processes, involving both specific and non-specific interactions with DNA (intercalation or groove binding and outside binding) and drug self-aggregation phenomena (Table 2).

**Figure 4:**
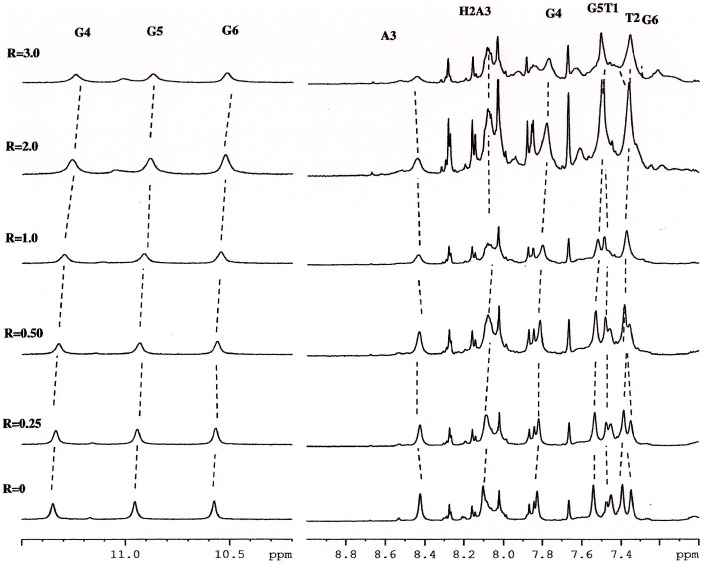
^1^H NMR spectra (11.5–10.2 ppm and 9.0–7.0 ppm) showing resonance of imino protons G4, G5 and G6 as well as the aromatic protons at different R = [2]/Htel.

On the other hand, our experience suggests that shift variations of oligonucleotide proton signals arise when a ligand intercalates between the base-pairs or binds to the minor groove [40], [41], [42]. The addition of the compound **1** to oligonucleotide solution induced progressively changes in the chemical shift of the DNA but only selected resonances are changed: *i.e.* NH imino and the aromatic proton of G4 (Δδ = −0.12 ppm and −0.11 ppm respectively), H1′ and H3′ of T2 (Δδ = −0.22 ppm and −0.19 ppm respectively). Moreover methyl of T1 (Δδ = +0.13 ppm), H3′ of T1 (Δδ = +0.17 ppm) experience a down field shift as well as aromatic protons of A3 and T2 (Δδ = +0.1 ppm). The other protons are almost unchanged and a very small up-field shift (Δ*δ ≤*0.1**ppm) was observed. In addition the oligonucleotide protons, specially, H8 of A3 became broad due to the complex formation (Figure 4). No separate signals were observed for the free and bound species, because an intermediate exchange, with respect to the NMR time scale, of the drug with the possible sites of binding of the oligonucleotide (Table 3).

**Figure 5:**
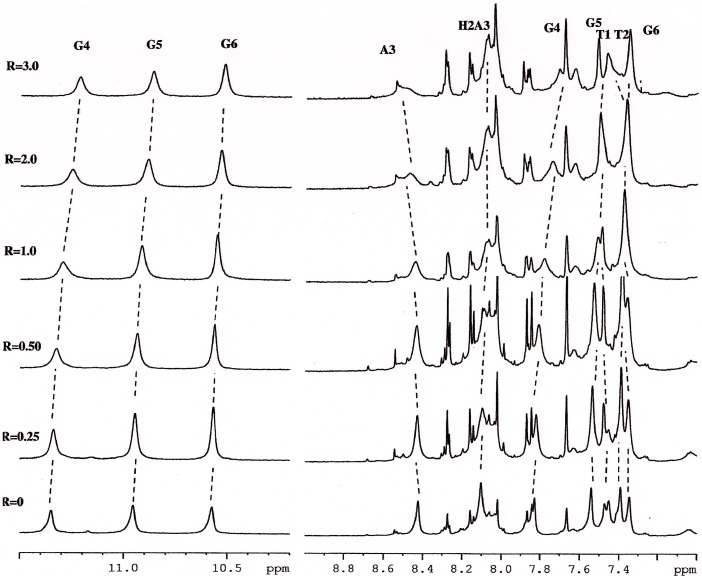
^1^H NMR spectra (11.5–10.2 ppm and 9.0–7.0 ppm), acquired at T = 25°C in H_2_O, containing 25 mM KH_2_PO_4_, KCl 150 mM and EDTA 1 mM (pH 6.7), showing resonance of imino protons G4, G5 and G6 as well as the aromatic protons at different R = [1]/Htel_._

The same experiments were performed with **2** (Figure 5). The results are the same even if, due to the extensive overlapping of **2** and oligonucleotide protons, the analysis was quite difficult. All the aromatic protons of ligand collapsed at 8.46 ppm, 7.88 ppm, 7.80 ppm and 7.38 ppm. Even in these case the addition of **2** to the oligonucleotide solution causes notable chemical shift variation on drug resonances, whereas the protons of TTAGGG are almost unchanged a part from an up-field shift of NH imino G4 and the aromatic proton of G4 (Δδ = −0.10 ppm), a down-filed shift of aromatic protons of T1, T2 and A3 (Δδ = +0.14 ppm, +0.11 ppm+0.12 ppm respectively), methyl of T1 and T2 (Δδ = +0.2 ppm and 0.12 ppm, respectively) (Table 3).

All these findings give a first indication of a potential intercalation binding mode of **1** and **2** near the T2A3G4 residues adjacent to the G-quadruplex quartets.

The ^1^H NOE experiments, allowing the detection of specific interactions between protons of the ligand and protons of the DNA, were performed in order to recognize possible preferred interaction sites. NOESY spectra were acquired with *R* = [drug]/[DNA] = 0.5 and 3.

**Figure 6:**
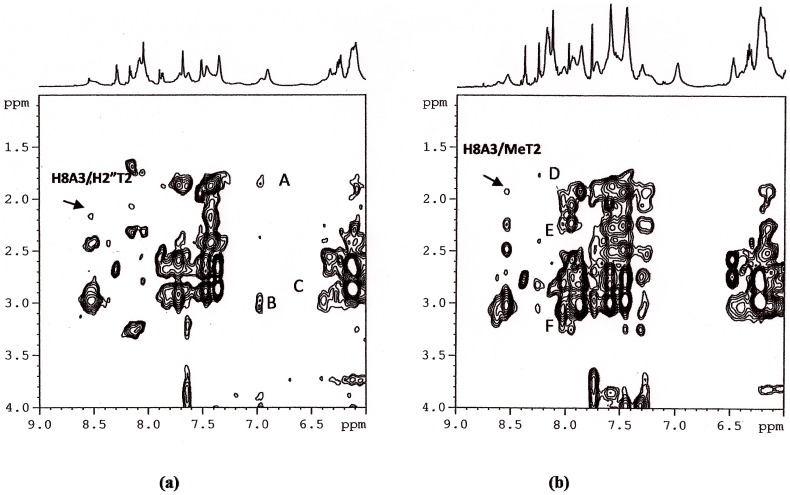
Selected region of 2D NOESY spectra of Htel/1 (a) and Htel/2 (b) complexes at 25°C in H_2_O, containing 25 mM KH_2_PO_4_, KCl 150 mM and EDTA 1 mM (pH 6.7). The weakness of H8A3/H2”T2 and of H8A3/MeT2 (total disappearance in the case of Htel/1) confirms a slight distortion at these level of the sequence. The peaks A, B and C are intermolecular NOEs, (A) H1 or H4 with CH_3_T2, (B) H1 or H4 with H2′, H2′′ A3, (C) H3 with H2′,H2′′A3, (D) Ar with MeT2 (E) Ar with H2′,H2′′ T2 (F) Ar with H2′,H2′′ A3.

The sequential NH imino cross peaks between G4, G5 and G6 are still observed in 2D NOESY of the complexes and it proves the position of the compound **1** between A3 and G4 without disrupting the G quartets. The presence of NOEs interactions, characteristic of the presence of G tetrads, together with the weakness of H8A3/H8G4, H8A3/H6T2, H8A3/H1′A3, H8A3/H2′′T2 and of H8A3/MeT2 (Figure 6(a) and (b)) (total disappearance in the case of the complex of **1** with Htel) in comparison with the oligonucleotide alone, confirms a slight distortion at these level of the sequence.

A certain number of NOE interactions between **1** and the DNA was extracted despite of some overlapping between the signals of TTAGGG and the **1** (Table 4). Examples are reported in Figure 6(a). The NOEs observed between the drug and the oligonucleotide protons provide information regarding the sequence of the binding sites and confirm that **1** prefers the A3G4 step of TTAGGG G-quadruplex as intercalation site with the indole positioned at A3T2 step. Specific intermolecular contacts were found, involving aromatic protons of indole moiety with the ribose protons of A3 and methyl of T2. For the acridine moiety, we detected NOE contacts with aromatic protons and H3′ of G4 and A3 residues. Following these few experimental NOEs, a model of the complex was built.

**Table 4:** Inter-molecular NOE interactions between protons of the 1 with protons of Htel^a^.

TTAGGG	1	Theoretical distances(Å)
CH_3_ T2	H1 or H4	4.31 (H4)
H2′ A3	H1 or H4	4.37 (H4)
H2′ A3	H2 or H3	4.33 (H3)
H1′ A3	H2 or H3	1.68 (H3)
H3′, G4 or A3	H3A	3.96 (A3)

The distances are calculated on the basis of the **1** best docked conformation.

a Acquired at 25°C, *R* = [drug]/[DNA] = 3. 2′ H and 2″ H stand for low field and up field proton respectively.

In the case of Htel and **2** complex it was very hard to unambiguously identify intermolecular interactions between **2** and DNA, due to the extensive overlapping, but a low number of NOE interactions was extracted from the 2D-NOESY and allowed to identify the **2** position inside the G-quadruplex structure. An aromatic proton (7.88 ppm) is close to the methyl, H2′ and H3′ of T2 unit and another aromatic protons (8.46 ppm) is close to H1′ and H2′A3 and G4. As for **1**, the interaction site for **2** is at the level of T2 A3 and G4 residues.

### NMR 9-amino Acridines-duplex Experiments

The dialysis experiments gave evidence that only the **1** interacts with the oligonucleotide double helix ds26. In order to better understand the specific interaction involved, we performed different NMR titration experiments. A *short* oligonucleotide, such as ds6, was used in previous studies with intercalating agents [40], [43], [44].

^1^H and ^31^P NMR titration experiments performed with **1** were fruitless: the addition of **1** to the double helix did not induce any chemical shield variation and did not induce line broadening of the oligonucleotide resonances similar to those observed with “classical” intercalating agents (Figure S2–S3) [40], [43], [44], [45]. These findings do not support the intercalation of **1** into the double helix.

The self-complementary oligomers ds8 and ds24 were used as models for CG- and AT-rich sequences respectively. These sequences are longer than the previous one and are partially contained into the ds26 sequence. In both cases NOESY experiments performed on the compound **1** and duplex did not show intermolecular interactions. Interestingly, the addition of **1** to a solution of the double helix fragments induces a line broadening of the resonances (Figure S4 and S5) of the oligomers. In particular the imino protons, that present almost unchanged chemical shift (Δδ <0.1 ppm) were observed to become very broad. The inverse titration experiment was performed with ds24 and with ds26 oligonucleotides (Figure S6 and S7, Table S2), by adding increasing amounts of DNA to a solution of **1** at constant concentration. No relevant chemical shift variations were detected, but a selective line broadening occurs for NH indole, aromatic 4A and 2A protons of 1 with ds26. These findings suggest a specific outside binding of **1** that can not happen with a shorter oligonucleotide as ds6 because of the hindrance of the side chains.

### Stability of 9-amino Acridines-Htel Complexes

**Figure 7:**
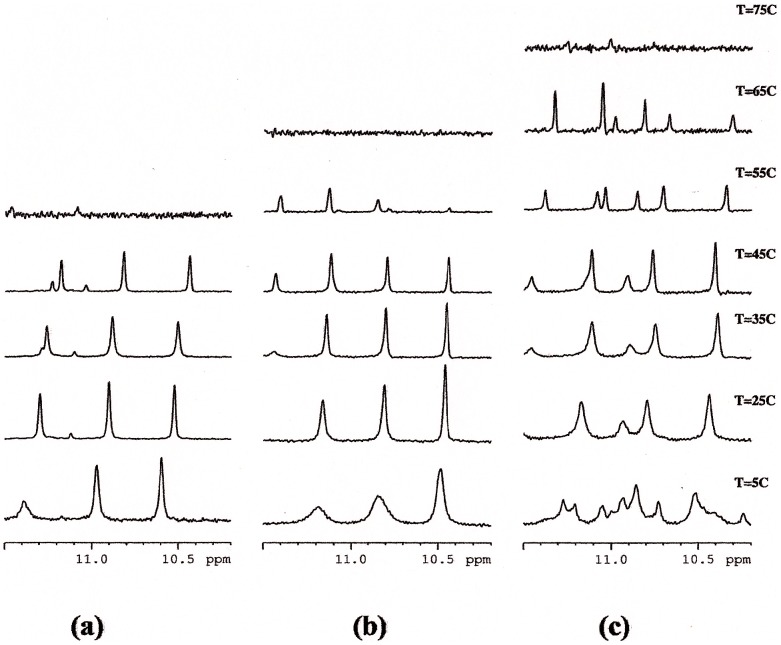
Imino protons regions of the NMR spectra of Htel (a) Htel/1(b) and Htel/2 (c) at different temperatures.

The imino proton region of the NMR spectra of both complexes clearly indicates the presence of G-quadruplex structure. Three imino signals are observed between 10.0 and 11.5 ppm (Figure 7 (a), (b), (c)). These spectra are consistent with a single G-quadruplex parallel structure similar to that of the Htel. In the case of Htel/**2** complex the exceeding number of imino signals in the NMR spectra of at 5°C suggests the presence of several species in equilibrium in slow exchange with respect to NMR time scale, although the chemical shifts variation were not significantly altered to consider a substantial change in topology.

We performed melting experiments in order to see whether **1** and **2** stabilize or not the G-quadruplex structure. The imino protons signals are diagnostic for the G-quadruplex formation and the melting of the structure causes their disappearance due to the break of the G quartet hydrogen bonds. The NH signals of Htel without drugs disappeared between 45°C and 55°C (Figure 7(a)) whereas in the presence of **1** and **2** they can still be observed at 55°C and disappear up to 55°C (Figure 7 (b,c)). These findings clearly indicate a significant stabilization of the G-quadruplex structure by the interaction with ligands. The slight higher stabilization induced by **2** compared with **1** are in agreement with more favorable specific interactions between different moieties of the ligand **2** to the DNA obtained by per residue energy free studies and discussed below.

### Model Generation

#### 9-amino acridines-HtelT G-quadruplex complexes

The 5′-TTAGGGT-3′ sequence, HtelT, was used as model for telomeric parallel G-quadruplex for the molecular docking of the ligands. In both cases, Autodock placed the ligand in an intercalated binding mode within the G-quadruplex. Docking experiments show that **1** fit in the original gap region, located between the virtual planes made by the four A and G bases, with the tryptophan group adjacent to A10 and T16, and the acridine moiety placed just under the A10 base, to give a π-π stacking interaction. It should be noted that the best docked orientation obtained for **1** has proven to be in agreement with experimental data of the inter-molecular NOE interactions previously discussed (as shown by the values of the distances reported in Table 4), thus, supporting the proposed binding model. On the contrary, compound **2** was unable to intercalate so efficiently, thus giving rise to a less stable orientation. The differences observed with respect to **1** could be due to the shift of the acridine moiety away from the center of the G-quadruplex, probably because of the greater steric hindrance produced by the presence of the piperidine group.

**Figure 8:**
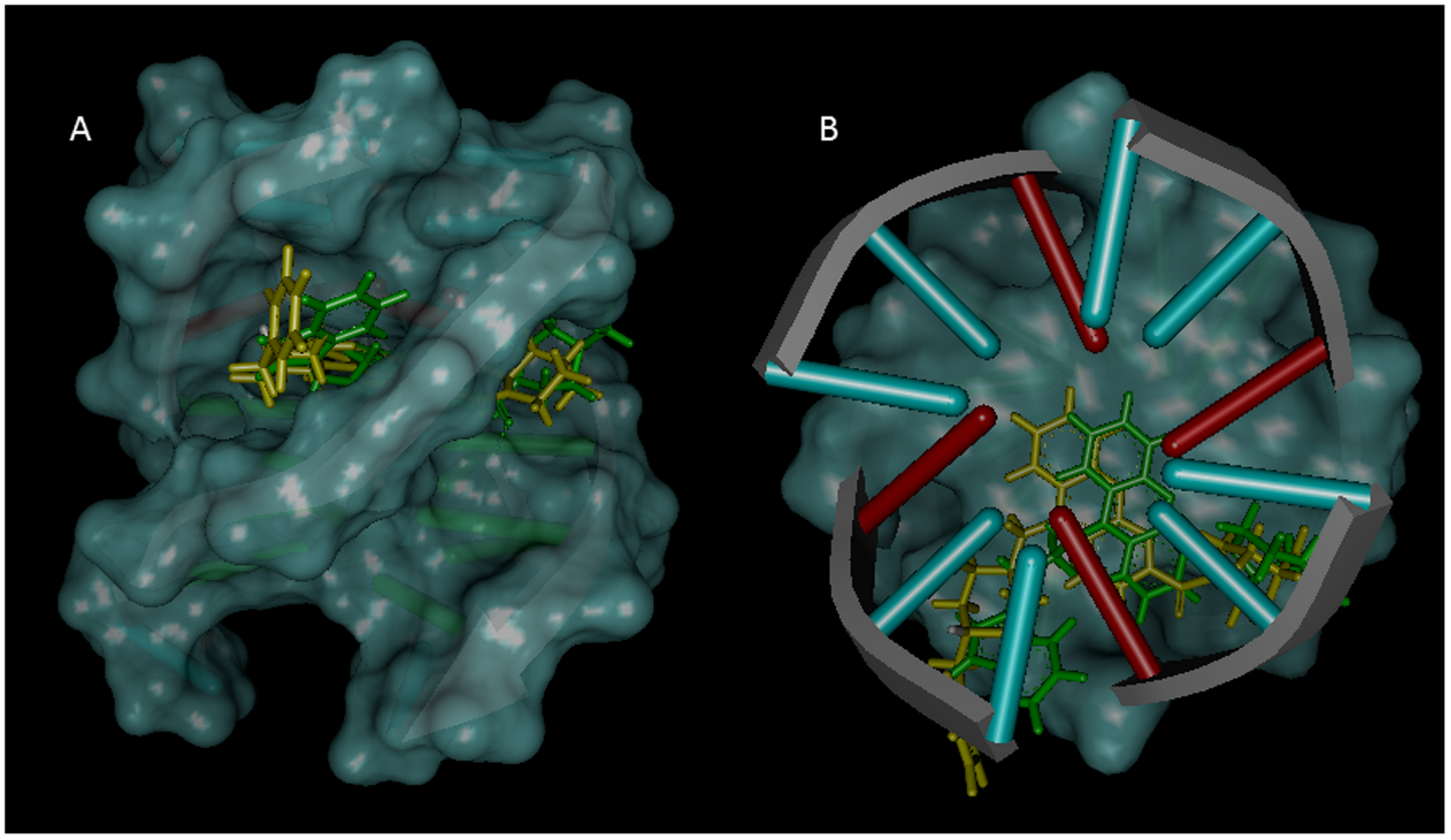
Lateral (A) and upper (B) views of the best docked conformations for 1 and 2. **1** is shown in green stick and **2** in yellow stick. In DNA, the base pairs are shown using the ladder representation, with the backbones displayed as arrows.

The above described systems were further optimized using the quantum mechanics/molecular mechanics (QM/MM) mixed approach. This technique allowed us to obtain a better and more complete description of the interactions with the G-quadruplex, as well as an estimate of the structural changes induced in the G-quadruplex by the binding of both ligands, **1** and **2**. In both cases, major differences were observed at the level of the acridine substituents, while the acridine ring maintained its position inside the G-quadruplex, together with the π-π interactions previously described (Figure 8 A and B). As we discuss below, molecular dynamic simulations support the proposed models based primarily on the observed NMR NOE’s and no other potential species were observed during the simulation.

**Figure 9:**
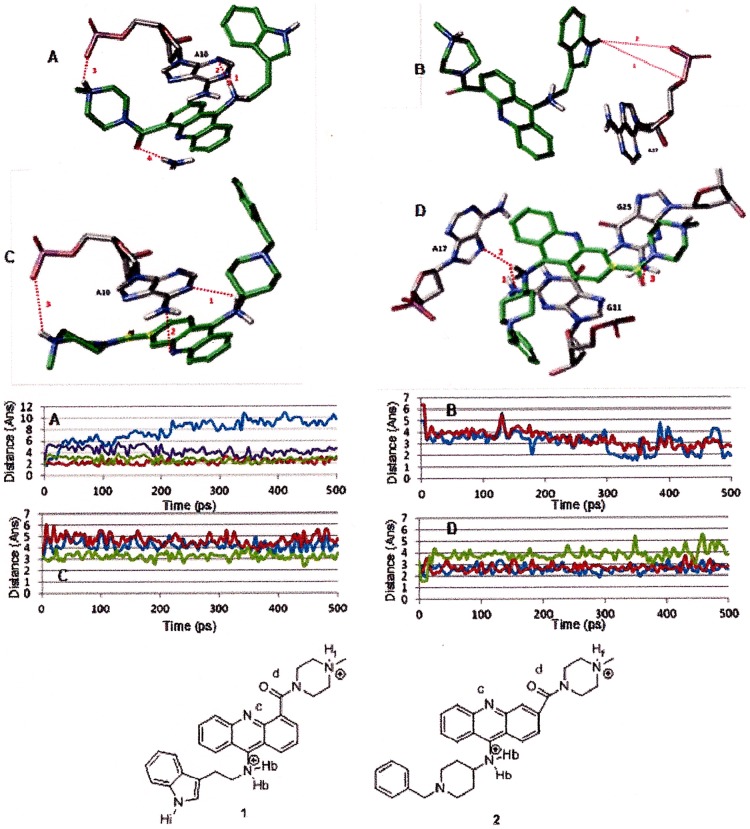
Time dependence of hydrogen bond distances observed between G-quadruplex and ligand 1 (A and B) and 2 (C and D). A)1. Hb and N1A10 (cyan) 2. Hb and N3A10 (green) 3. Hf and OPA10 (blue) 4. O_D_ and HN2G25 (red). B) 1. Hi and OaA17 (blue) 2. Hi and OPA17 (red). C) 1. Hb and N1A10 (blue) 2. Nc and HN6A10 (red) 3. Hf and OPA10 (green) D) 1. Hb and N2G11 (blue) 2. Hb and N7A17 (red) 3. Od and HN2G25 (green).

The position of compound **1** is stabilized by four strong hydrogen bonds: two between the 9-amino acridine hydrogen and N1A10 and N3A10 of 1.8 and 2.6 Å, other between the methyl piperazine hydrogen and OPA10 of 1.95 Å and other between the carbonyl oxygen and HN2G25 of 2.12 Å (Figure 9 A). Considering the structure of the G-quadruplex, the presence of **1** has influenced mainly the A10 position. Indeed due to the presence of the ligand, A10 undergoes a small clockwise rotation (about 6°) with respect to the original structure. This rotation of A10 leads to the formation of an additional hydrogen bond with T9, while keeping the two original hydrogen bonds with A24 and A17.

In the case of **2** an orientation close to that of **1** was obtained. This position is stabilized by six hydrogen bonds: three between the 9-amino acridine hydrogen and N1A10, N2G11 and N7A17 of 3.1, 2.3 and 2.8 Å; other between the methyl piperazine hydrogen and OPA10 3.3 Å; other between the carbonyl oxygen and HN2G25 of 3.3 Å and other between the acridine nitrogen and HN6A10 of 3.5 Å (Figure 9 C and D).

However, the biggest difference between **1** and **2** was found to be at the level of their interaction with the G-quadruplex. Differently from **1**, the interaction of **2** resulted in a clockwise rotation of G11 (about 9°) with respect to the original structure. This rotation allowed G11 to form two new hydrogen bonds with G26, while losing two of the four hydrogen bonds with G18 and G25.

### MD Simulation

Computational methods are widely used to investigate biomolecules and complexes, and have been shown to provide valuable deeper understanding of the structural, dynamic and energetic properties [46], [47], [48].

**Figure 10:**
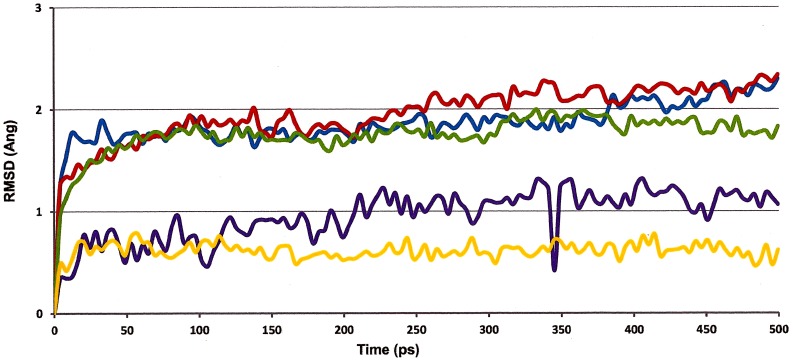
Time dependence of the RMSD of ligand 1 and 2 on the complex (cyan and yellow, respectively) and A and G-quartet heavy atoms (blue) with ligand atoms (1 and 2 are shown in red and green, respectively) at 298 **K.**

To assess the overall structural stability of the 9-amino acridines – G-quadraplex complex, we evaluated the root mean square deviation (rmsd) of the whole structure over the course of the MD simulation. G-quadruplex structures without potassium between the G-tetrads were structurally less stable than K^+^ saturation complexes and significant disorder was observed during the MD simulation (results not shown). The rmsd values of the whole complex remain <4 Å (Figure S8) reflecting the fluctuations of the terminal T residues, as they are not held tightly by hydrogen bonds and, hence, are free to move during simulation. To consistently examine the stability of the complex without the interference of the terminal residues, the rmsd values for the A and G-quartets core, with bound ligand **1** and **2**, were examined and their structural integrity were conserved in both cases with less than 2.5 Å rmsd (Figure 10). To examine how 9-amino acridines occupies within the A and G tetrad, we evaluated the rmsd values for the ligands **1** and **2** within the G-quadruplex, showing that acridine scaffolds are free to move within the gap region between the virtual planes made by the four A and G bases, but remains within the G-quadruplex (Figure 10).

To examine the overall stability of the structural complex, MD simulations at elevated temperature were also performed. The goal was to identify the specific interactions observed in our NMR studies that can play an important role in maintaining the G-quadruplex structure. We expect transient interactions will be subsided at elevated temperature allowing us to identify the stable contacts essential for retaining the overall G-quadruplex structure. The rmsd values for these studies are shown in Figure S9–S10. The MD simulation at 400 K indicates that the rmsd values for the A and G-quartets core with bound ligand **2** is similar to value obtained at 298 K. In the case of ligand **1**, the higher value of rmsd indicates that interactions established between ligand and HtelT were not maintained at high temperature (Figure S9). Significant disorder was observed during the simulation at 500 K, only G-quadruplex with bound ligand **2** keep the G-quadruplex structure as indicated the rmsd value for the A and G-quartets core (Figure S10). This suggests that ligand **2** has a more stabilizing effect on the quadruplex structure as compared to **1**.

We also examined the essential hydrogen bonding for 9-amino acridines binding. An interaction was considered to be a hydrogen bond if the distance between the hydrogen donor and acceptor was less than 3.5 Å. As mentioned above four hydrogen bounds are formed with ligand **1**, only the bond between the methyl piperazine hydrogen and OPA10 was not kept during the dynamic simulation due to the rotation of the N-Me-Piperazine ring. Moreover, during the simulation the rotation of the indole group has allowed the formation of two additional hydrogen bonds between NH and OA17 and OPA17 from 6.4 and 5.0 A to 2.7 and 1.9 Å (Figure 9B). In the case of ligand **2**, six hydrogen bounds are observed, but weaker compared with hydrogen bonds formed with ligand **1**. The hydrogen bond between 9-amino acridine hydrogen and N2G11 and N7A17 and between N-Me piperazine hydrogen and OPA10 were kept during the simulation (Figure 9C and D).The N-Bz-piperidine group acts as the indole in **1** but, probably due to the bulkiness of the phenyl group, in this case the quaternary nitrogen fails to approach the phosphate group of A17 enough to form hydrogen bonds.

**Figure 11:**
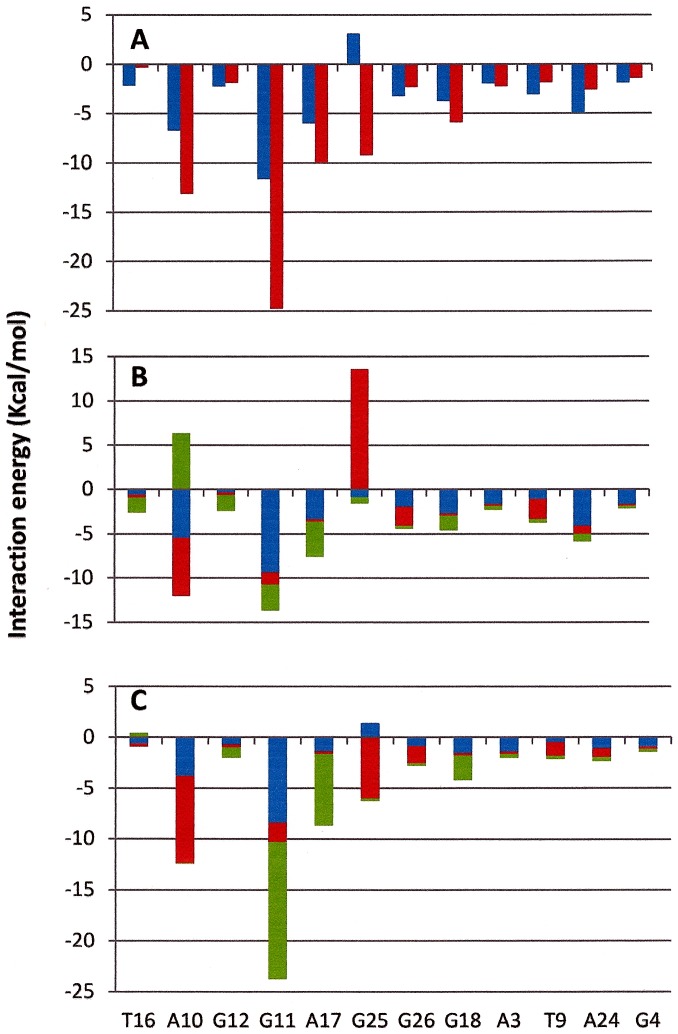
Per residue interaction energy in Kcal/mol between compound 1 (blue) and 2 (red) (A), between acridine (blue), piperazine (red) and tryptophan (green) moiety in compound 1 (B) and between acridine (blue), piperazine (red) and piperidine (green) in compound 2 (C).

Finally, the interaction energy per residue between compound **1** and **2** to individual nucleotide residues of HtelT G-quadruplex are shown in Figure 11. Examination of the interactions revealed a slight variation in the signature of binding between compounds **1** and **2** with the G-quadruplex DNA (Figure 11A). The main difference is found in the G25. Figure 10 B-C show the interaction energy per residue between moieties that form compound **1** and **2**. While as piperazine moiety in compound **2** has an energetic favorable contribution, in compound **1** this interaction is not favorable. The slight higher HtelT stabilization induced by compound **2** observed during NMR melting experiments may be due to the most favorable interactions between the different moieties of **2** with the G-quadruplex.

### Conclusions

In summary we have used fluorescence titration assays, competitive dialysis, NMR studies and molecular dynamics simulations in order to determine the binding properties of preclinical 9-amino acridines to DNA. A selectivity of acridine derivatives for G-quadruplex structures was observed. Slightly higher stabilization of the structural complex induced by the interaction of compound **2** as compared to **1** was observed by NMR melting experiments. Detailed structural studies by NMR and molecular dynamic simulation on G-quadruplex telomeric complex showed the core of both 9-amino acridines intercalates directly between the virtual planes made by the four A and G bases via π- π interactions, but do not exactly overlap. The indole moiety in ligand **1** does not fit as closely to the G-quadruplex groove as the N-Bz-piperidine group in ligand **2**. Structurally, ligand **1** establishes only four strong hydrogen bonds with telomeric G-quadruplex while ligand **2** established six. The most significant interaction observed involved the carbonyl oxygen with G25 for both ligands and the 9-amino acridine hydrogen with A10 for ligand **1** and with A17 and G11 for ligand **2**. Per residue interaction free energy profiles of each compounds showed the substituents of ligand **1** exhibit two distinctly unfavorable interactions between the piperazine group to G25 and the piperidine group to A10 (Figure 11B). The strength of these interactions was further examined by MD simulations at elevated temperatures showing the interactions between ligand **2** and the G-quadruplex are tighter than that of ligand **1**. Rmsd analysis over the course of simulation further support the slight enhanced structural stabilization by compound **2** over compound **1** with relative lower rmsd among the unbound and bound complexes. The slightly stronger interactions between ligand **2** to the G-quadruplex over ligand **1** could explain the experimental differences in structural stabilities between the two 9-amino acridines. The ability of 9-amino acridines to exhibit similar binding affinity to the G-quadruplex while inducing different level of structural stabilization through intercalation could be a unique strategy for altering the overall biological function of telomerase and their subsequent anticancer activity. These findings will assist in the understanding the parameters influencing the G-quadruplex – ligand interaction and will serve as an enhanced platform for rational drug design.

## Materials and Methods

### Compounds and Oligonucleotide Synthesis

Compounds **1** and **2** were synthesized according to published procedures [27]. All the standard phosphoroamidites and reagents for DNA synthesis were purchased from Applied Biosystems and from Link Technologies. The synthesis of the oligonucleotides was performed at 1 µmol scale on an Applied Biosystems’ DNA/RNA 3400 synthesizer by solid-phase 2-cyanoethylphosphoroamidite chemistry. The studied sequences are listed in table 1. The resulting oligonucleotides were purified by HPLC and desalted in a Sephadex (NAP-10) G25 column.

### Competitive Dialysis Studies

A 100 µl of a 50 µM oligonucleotide in potassium phosphate buffer was introduced into a separated dialysis unit and a blank sample containing only buffer. All dialysis units were allowed to equilibrate during 24 h at room temperature in a beaker containing the 1 µM solution of the appropriate acridine derivative. At the end of the dialysis experiment, the amount of ligand bound to the DNA was quantified by fluorescence after the digestion of the oligonucleotide (λ_ex_ and λ_em_ were set to 265 nm and 435 nm, respectively) [49].

### NMR Spectroscopy

The NMR spectra were recorded by Bruker AV-600 spectrometer operating at a frequency of 600.10 MHz for ^1^H and 242.94 MHz for ^31^P nuclei, equipped with a *z*-gradient triple resonance TXI and 5 mm BB probe. ^1^H and ^31^P spectra (broad-band ^1^H decoupled mode) were recorded at variable temperature ranging from 5°C to 75°C. Chemical shifts (δ) were measured in ppm. ^1^H and ^31^P NMR spectra were referenced respectively to external DSS (2,2-dimethyl-2-silapentane-5-sulfonate sodium salt) set at 0.00 ppm and MDA (methylenedisphosphonic acid) set at 16.8 ppm. Estimated accuracy for protons is within 0.01 ppm, for phosphorous is within 0.03 ppm.

Standard homonuclear 2D-NMR experiments were performed to assign the resonances of the complexes, including DQF-COSY, TOCSY and NOESY [50], [51]. The mixing times were set at 150 ms and 300 ms for NOESY and 60 ms for TOCSY. For samples in H_2_O, the excitation sculpting sequences from standard Bruker pulse program libraries were employed. Typically, 2048×1024 data points were acquired using TPPI and transformed to a final 4 K×4 K real data matrix after apodisation with a 90° and 90°-shifted sine-bell squared function in f2- and f1-domain, respectively. Baseline correction was achieved by a 5th-degree polynomial function. ^1^H assignments for ligands were performed by using ROESY (spin lock 300 ms) and TOCSY experiments. The sequential assignments in free and bound oligonucleotides were performed by applying well established procedures for the analysis of double stranded and quadruplex structures. The program Sparky [52] was used to assign the NOESY cross-peaks. The G-quadruplex Htel and duplexes ds6, ds7 and ds32 were previously assigned [39], [40].

The samples for NMR measurements were dissolved in 500 µl H_2_O/D_2_O (9∶1) containing 25 mM KH_2_PO_4_, KCl 150 mM and EDTA 1 mM (pH 6.7) for the G-quadruplex Htel and containing 10 mM KH_2_PO_4_, KCl 70 mM and EDTA 0.2 mM (pH 7.0) for the double helix ds6, ds7, ds26 and ds24. The final concentration of the oligonucleotides was ranging between 0.2–0.7 mM. A stock solution of **1** and **2** was prepared in DMSO-d6 at the concentration of 20 mM.

NMR titration was performed by adding increasing amounts of **1** and **2** to the oligonucleotides solution at R = [Ligand]/[DNA] ratio equal to 0, 0.25, 0.5, 0.75, 1, 2 and 3 and in inverse order, by adding increasing amounts of DNA to a solution of **1** from R = 40 to R = 1.0.

### Fluorescence Assays

The study of the interaction equilibrium of **1** and **2** and the G-quadruplex Htel or the duplex ds6 consists of recording the fluorescence spectra of a 1 µM solution of the drug after the addition of increasing amounts of oligonucleotide (from 0 to 25 µM) in potassium phosphate buffer (185 mM NaCl, 185 mM KCl, 6 mM Na_2_HPO_4_, 2 mM NaH_2_PO_4_, 1 mM Na_2_ EDTA at pH 7).

The emission spectra of the resulting solutions were recorded from 300 to 500 nm at 265 nm excitation wavelength at 25C. The macroscopic binding constant corresponding to complex formation was calculated from the multivariate analysis of fluorescence data recorded in the range 300–425 nm using the hard modeling program Equispec [53].

### Molecular Modeling

The model was built based on a G-quadruplex NMR structure 5′-TTAGGGT-3′ HtelT, in complex with a quinacridine-based ligand (N,N’-(dibenzo[b,j] [1], [7]phenanthroline-2,10-diyldimethanediyl)dipropan-1-amine) (PDB code 2JWQ) [46]. After the separation of the coordinates of ligands and DNA, polar hydrogens were added with the GROMACS package [54] using the GROMOS 53a6 force field [55].The structures of **1** and **2** were refined using a systematic conformer, search followed by geometry optimization of the lowest energy structure with MOPAC (PM3 Methods, RMS gradient 0.0100) [56].

Molecular docking experiments were performed with Autodock 4.0, which uses an empirical scoring function based on the free energy of binding [57], [58]. The 9-aminoacridines (**1** and **2**) and the DNA G-quadruplex were further processed using the Autodock Tool Kit (ADT) [59]: Gasteiger-Marsili charges [60] were assigned to **1** and **2** and Cornell parameters were used for the phosphorous atoms in the DNA. Solvation parameters were added to the final docked structure using Addsol utility. Structures with less than 1.0 Å root-mean-square deviation (rmsd) were clustered together and representative model of each cluster was selected based on the most favorable free energy of binding. Visual inspection was carried out to select the final structure with the expected mode of intercalation, minor groove binding, or others (major groove binding, interaction with phosphate groups, etc.).

In the current study, we used the pseudo-bond *ab-initio* QM/MM approach as implemented in Gaussian-03 [61]. For the QM/MM calculations, the DNA-ligand system resulting from the docking study was first partitioned into a QM subsystem and an MM subsystem. The reaction system used a smaller QM subsystem consisting of the ligand and bases within 3.5 Å, whereas the rest of the system (the MM subsystem) was treated using the AMBER force field, together with a low memory convergence algorithm. The boundary problem between the QM and MM subsystems was treated using the pseudo-bond approach. With this G-quadruplex-substrate QM/MM system, an iterative optimization procedure was applied to the QM/MM system, using B3LYP/3-21G* QM/MM calculations, leading to an optimized structure for the reactants. The convergence criterion used was set to obtain an energy gradient of <10^−4^, using the twin-range cutoff method for nonbonded interactions, with a long-range cutoff of 14 Å and a short-range cutoff of 8 Å.

### MD Simulation

All simulations were carried out using IMPACT (New York, NY) with the OPLS2005 force field [62] and the TIP3P water model [63] at 298, 400 and 500 K. Two potassium ions were manually overlayed into the central channel between the G-quartet planes in the complex models. Each DNA complex was solvated in a rectangular box with a 10 Å water buffer from the DNA. Na^+^ and Cl^−^ counterions were added at 5 Å from the box boundary to neutralize the total charge of the system.

Each system was initialized by a 1000-step conjugate gradient energy minimization. The simulations were carried out under the periodic boundary condition using particle mesh Ewald [64]. The SHAKE method [65] was employed to restrain all hydrogen bonds. Atoms involved in NOE’s bonds were restrained to their experimental value at (500 kcal/mol Å). Snapshots of the simulated trajectories were collected at 1 ps time intervals.

## Supporting Information

Figure S1 **Fluorescence titration spectra.** Fluorescence spectra of a 1 µM solution of **1** (left) and **2** (right) after the addition of increasing amounts of Htel (from 0 to 25 µM) in potassium phosphate buffer. Excitation wavelength is 265 nm.
(TIF)

Figure S2 **^1^H NMR spectra (15–12 ppm and 9.0–5.5 ppm) showing resonance of imino and aromatic and ribose H1′ protons region at different R =** [**1**] **/[ds6]_._**
(TIF)

Figure S3 **^1^H decoupled ^31^P NMR spectra of (a) ds6 at T = 25°C, (b) R =** [**1**] **/[ds6] = 3.0.**
(TIF)

Figure S4 **^1^H NMR spectra (15–12 ppm and 10–5.5 ppm) showing resonance of imino and aromatic and ribose H1′ protons region at different R =** [**1**] **/[ds8].**
(TIF)

Figure S5 **^1^H NMR spectra (15–12 ppm and 10–5.5 ppm) showing resonance of imino and aromatic and ribose H1′ protons region at different R =** [**1**] **/[ds24].**
(TIF)

Figure S6 **^1^H NMR spectra showing NH of indole moiety and aromatic protons of 1 in the free state (R = [ds24]/** [**1**] **= 0) and at different R.** High and low R values must be related to the free and bound state of DMF1 in solution respectively.
(TIF)

Figure S7 **^1^H NMR spectra showing NH of indole moiety and aromatic protons of 1 in the free state (R = [ds26]/** [**1**] **= 0) and at different R.** High and low R values must be related to the free and bound state of **1** in solution respectively.
(TIF)

Figure S8 **Time dependence of the RMSD of heavy atoms of complete G-quadruplex (blue) with ligand atoms (1 and 2 are shown in red and green, respectively) at 298 K.**
(TIF)

Figure S9 **Time dependence of the RMSD of ligand 1 and 2 on the complex (cyan and yellow, respectively) and A and G-quartet heavy atoms (blue) with ligand atoms (1 and 2 are shown in red and green, respectively) at 400** **K.**
(TIF)

Figure S10 **Time dependence of the RMSD of ligand 1 and 2 on the complex (cyan and yellow, respectively) and A and G-quartet heavy atoms (blue) with ligand atoms (1 and 2 are shown in red and green, respectively) at 500** **K.**
(TIF)

Table S1 **Logarithm of the binding constants calculated using Equispec program. n.d. not determinded due to lack of changes in the fluorescence spectra.**
(DOCX)

Table S2 **Table S2. Selected ^1^H chemical shift assignments for ds24 (δ) in the presence of 1** ***^a^***.
(DOCX)
