# Correction: Structure and Stability of Human Telomeric G-Quadruplex with Preclinical 9-Amino Acridines

**DOI:** 10.1371/annotation/e49600ba-2cfa-45c6-9984-2b337ad73add

**Published:** 2013-12-05

**Authors:** Ruben Ferreira, Roberto Artali, Adam Benoit, Raimundo Gargallo, Ramon Eritja, David M. Ferguson, Yuk Y. Sham, Stefania Mazzini

This article was republished on December 3rd, 2013 because of incorrect figure order. Please download this article again to view the figures. 

