# Supplementary figures and images for: Structure and Stability of Human Telomeric G-Quadruplex with Preclinical 9-Amino Acridines
Source: PLoS One. 2013 Mar 15;8(3):e57701. doi: 10.1371/journal.pone.0057701 (PMC3598906; doi:10.1371/journal.pone.0057701)

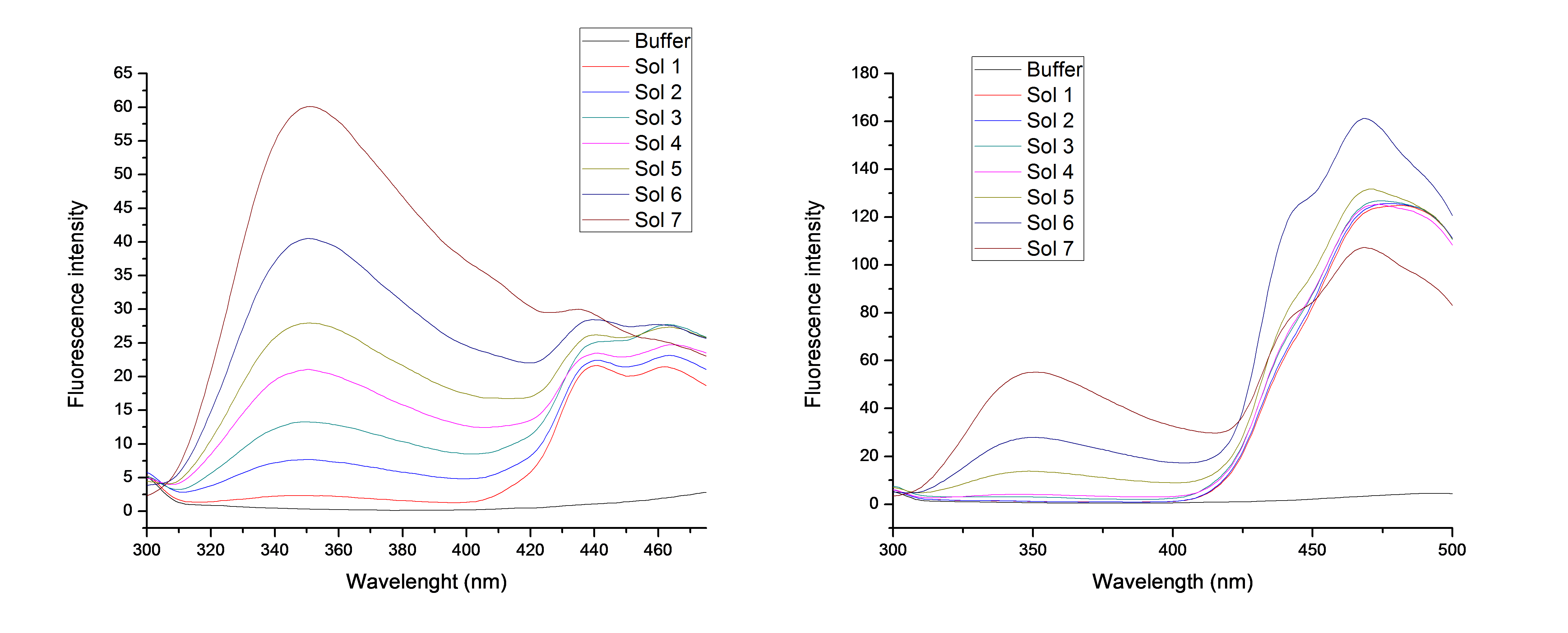

Supplement: Figure S1 — Fluorescence titration spectra. Fluorescence spectra of a 1 µM solution of 1 (left) and 2 (right) after the addition of increasing amounts of Htel (from 0 to 25 µM) in potassium phosphate buffer. Excitation wavelength is 265 nm. (TIF) [file pone.0057701.s001.tif]

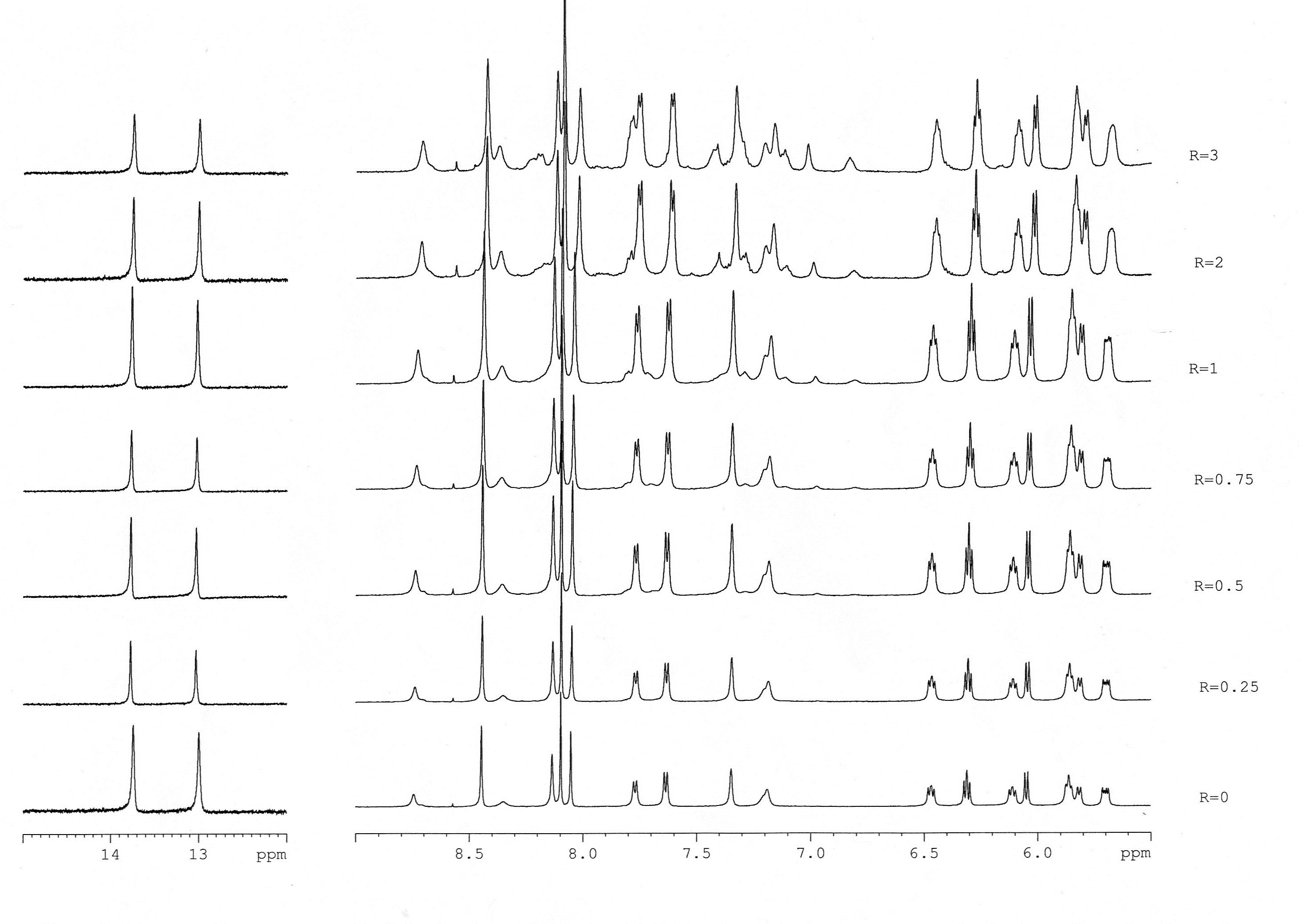

Supplement: Figure S2 — 1H NMR spectra (15–12 ppm and 9.0–5.5 ppm) showing resonance of imino and aromatic and ribose H1′ protons region at different R = [1] /[ds6]. (TIF) [file pone.0057701.s002.tif]

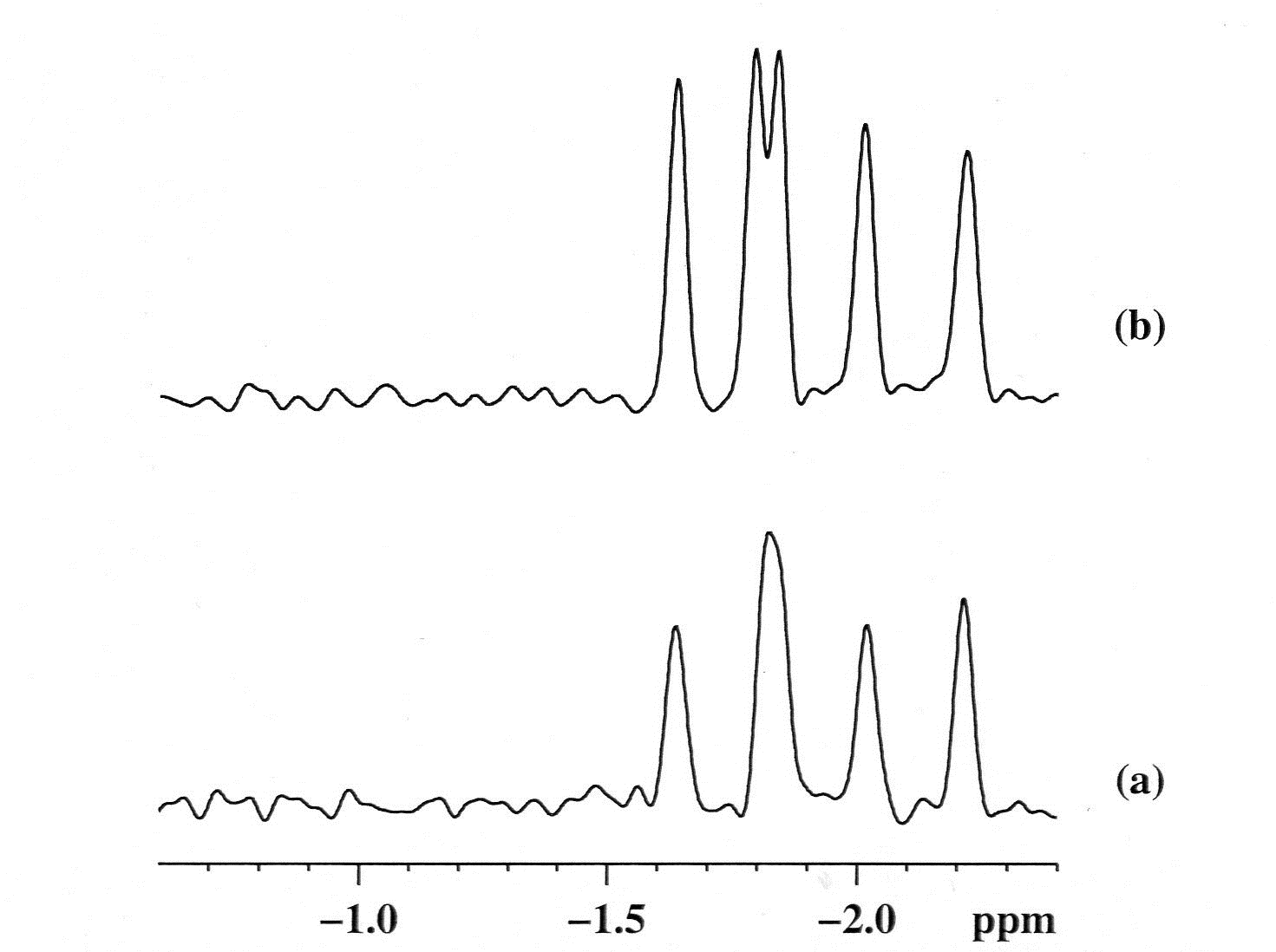

Supplement: Figure S3 — 1H decoupled 31P NMR spectra of (a) ds6 at T = 25°C, (b) R = [1] /[ds6] = 3.0. (TIF) [file pone.0057701.s003.tif]

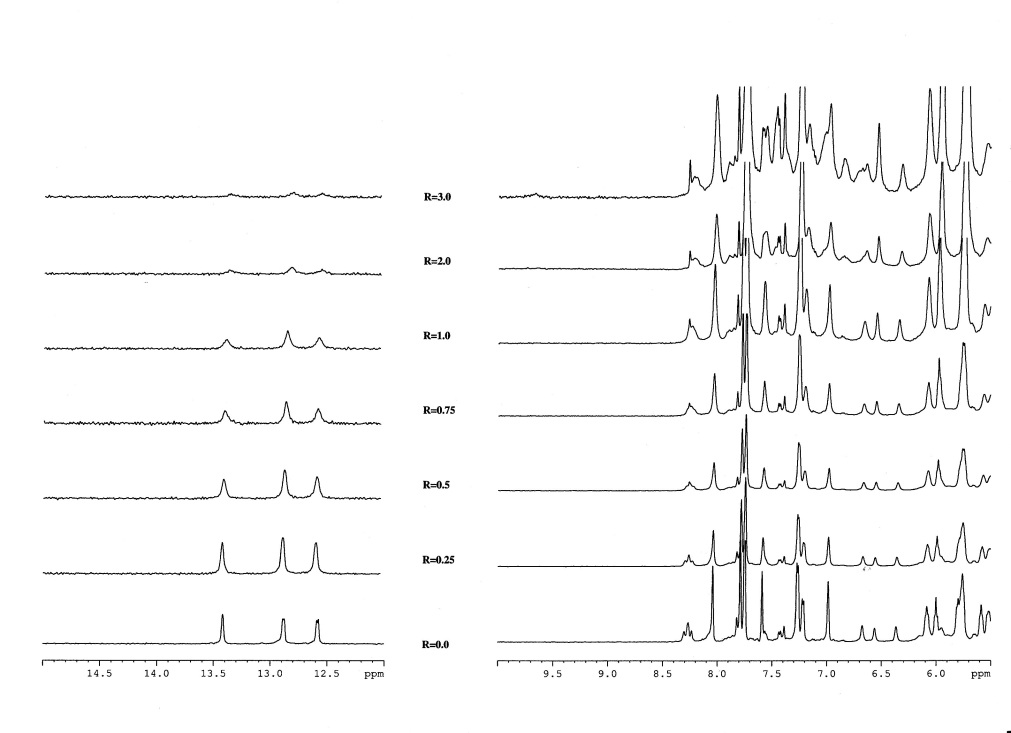

Supplement: Figure S4 — 1H NMR spectra (15–12 ppm and 10–5.5 ppm) showing resonance of imino and aromatic and ribose H1′ protons region at different R = [1] /[ds8]. (TIF) [file pone.0057701.s004.tif]

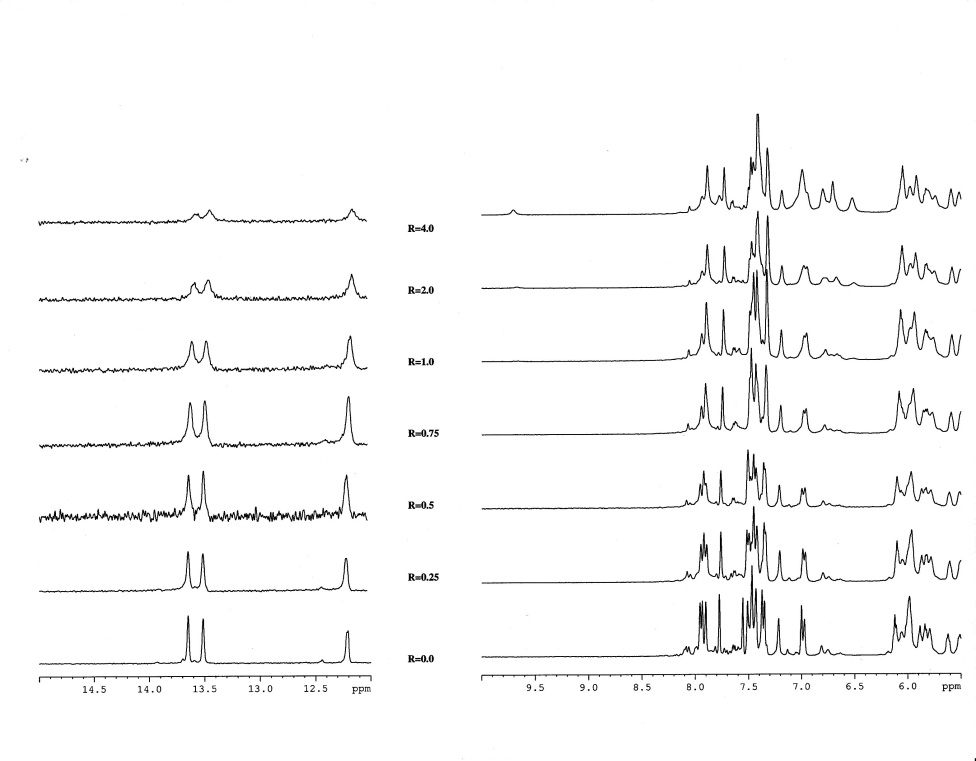

Supplement: Figure S5 — 1H NMR spectra (15–12 ppm and 10–5.5 ppm) showing resonance of imino and aromatic and ribose H1′ protons region at different R = [1] /[ds24]. (TIF) [file pone.0057701.s005.tif]

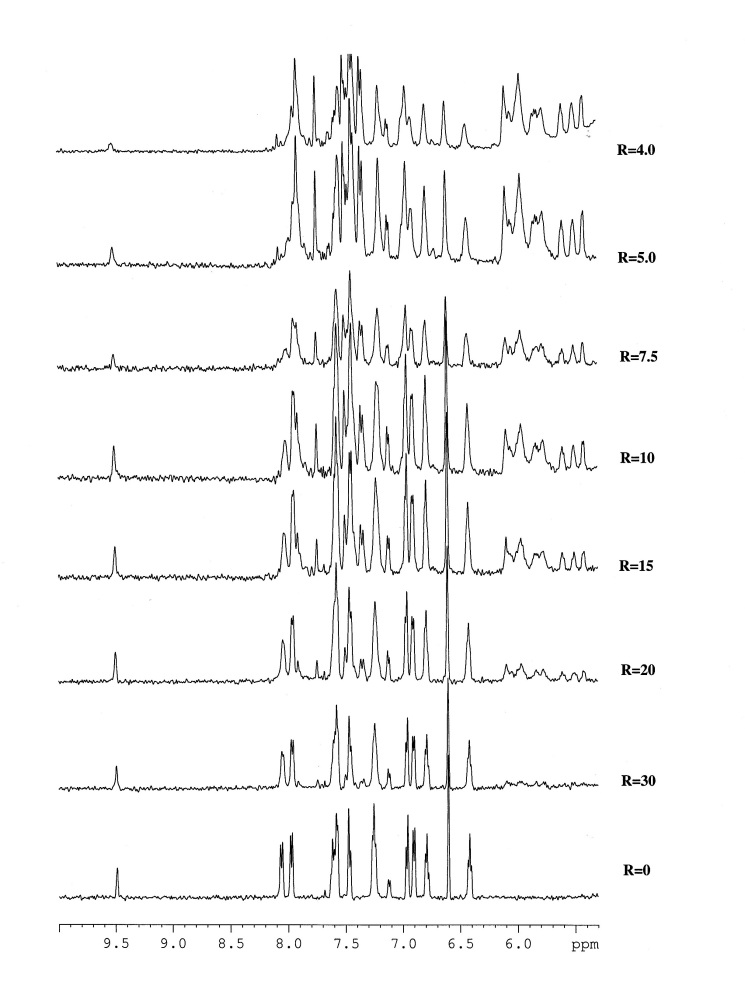

Supplement: Figure S6 — 1H NMR spectra showing NH of indole moiety and aromatic protons of 1 in the free state (R = [ds24]/ [1] = 0) and at different R. High and low R values must be related to the free and bound state of DMF1 in solution respectively. (TIF) [file pone.0057701.s006.tif]

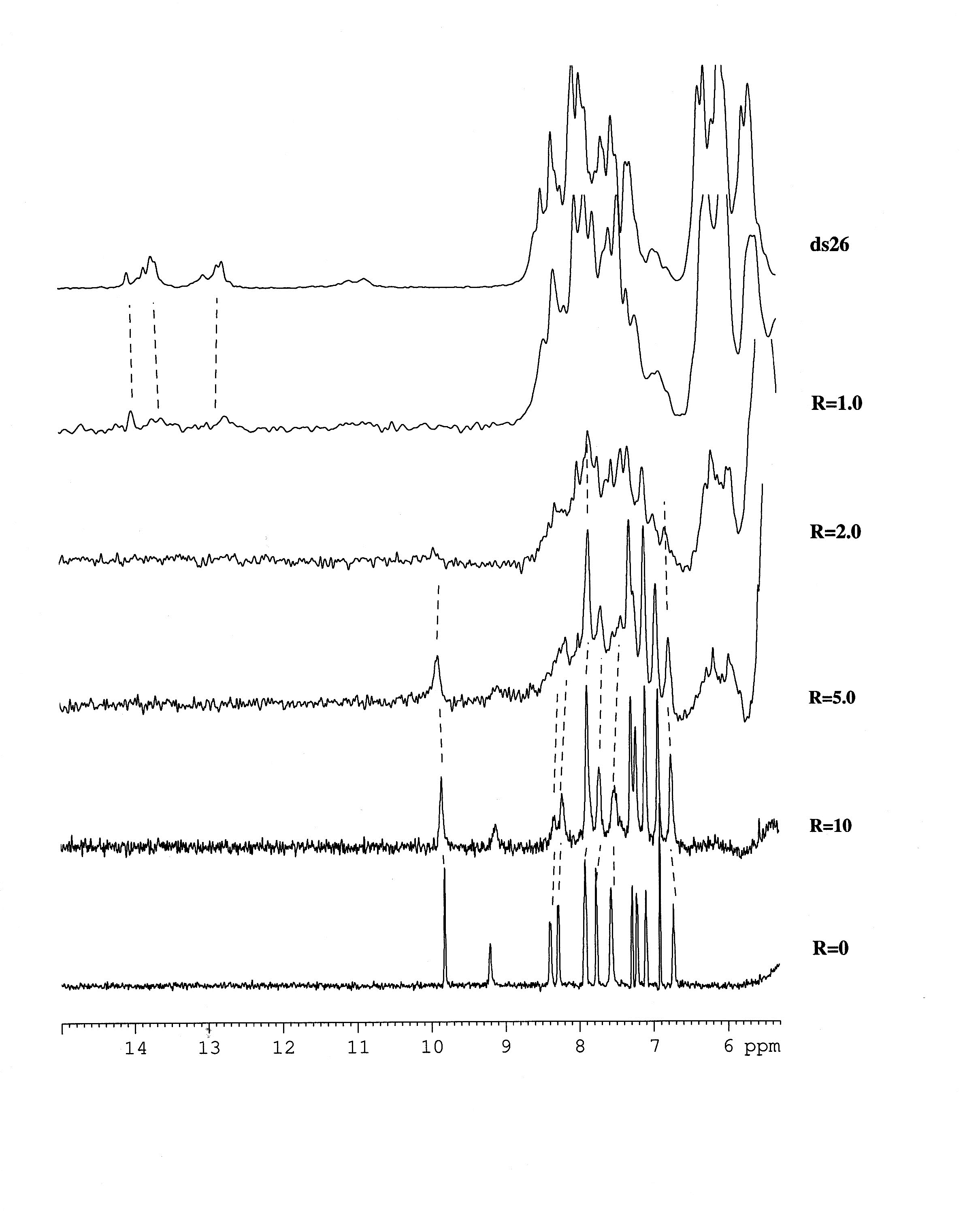

Supplement: Figure S7 — 1H NMR spectra showing NH of indole moiety and aromatic protons of 1 in the free state (R = [ds26]/ [1] = 0) and at different R. High and low R values must be related to the free and bound state of 1 in solution respectively. (TIF) [file pone.0057701.s007.tif]

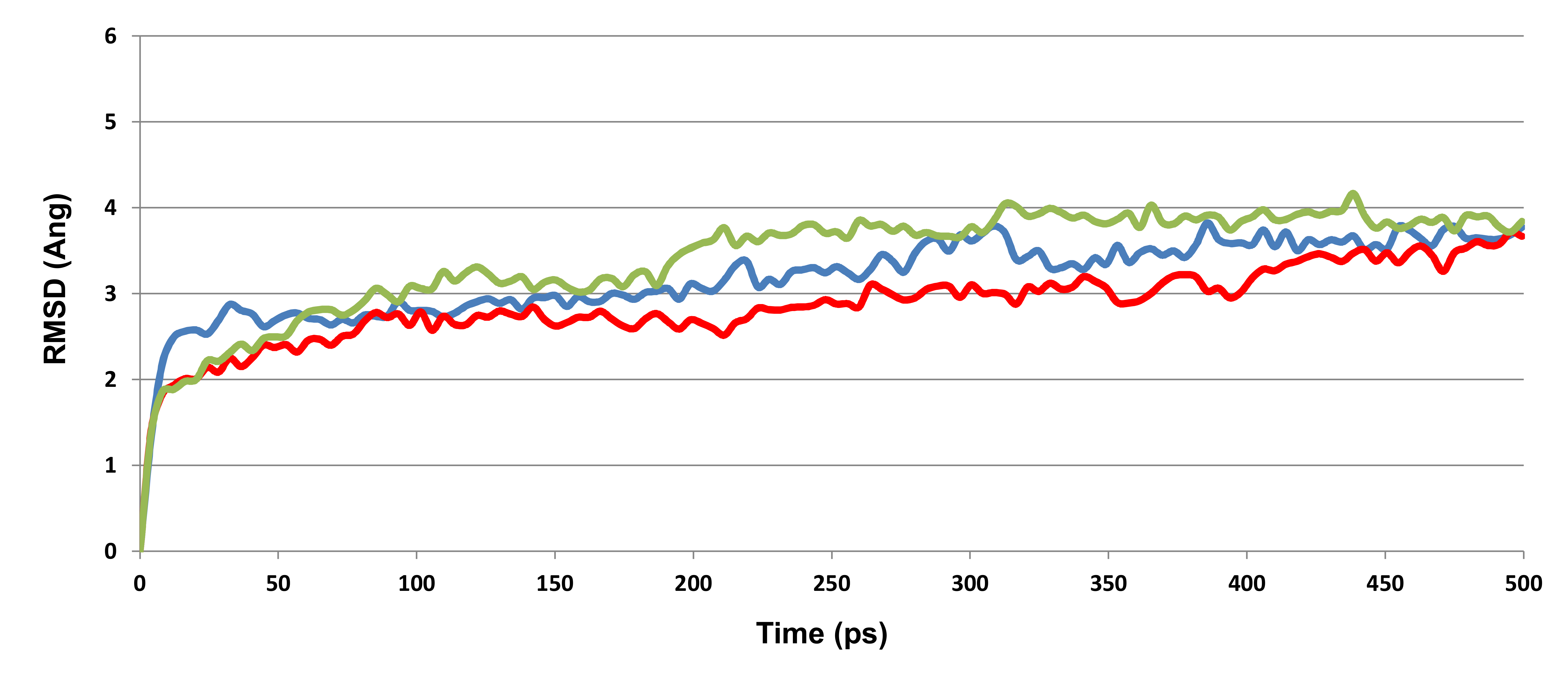

Supplement: Figure S8 — Time dependence of the RMSD of heavy atoms of complete G-quadruplex (blue) with ligand atoms (1 and 2 are shown in red and green, respectively) at 298 K. (TIF) [file pone.0057701.s008.tif]

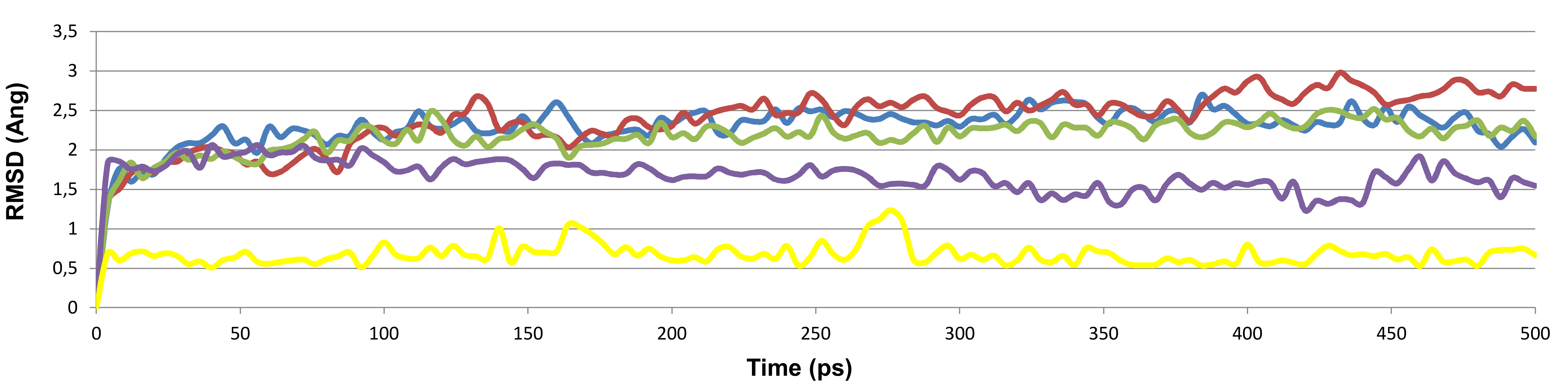

Supplement: Figure S9 — Time dependence of the RMSD of ligand 1 and 2 on the complex (cyan and yellow, respectively) and A and G-quartet heavy atoms (blue) with ligand atoms (1 and 2 are shown in red and green, respectively) at 400 K. (TIF) [file pone.0057701.s009.tif]

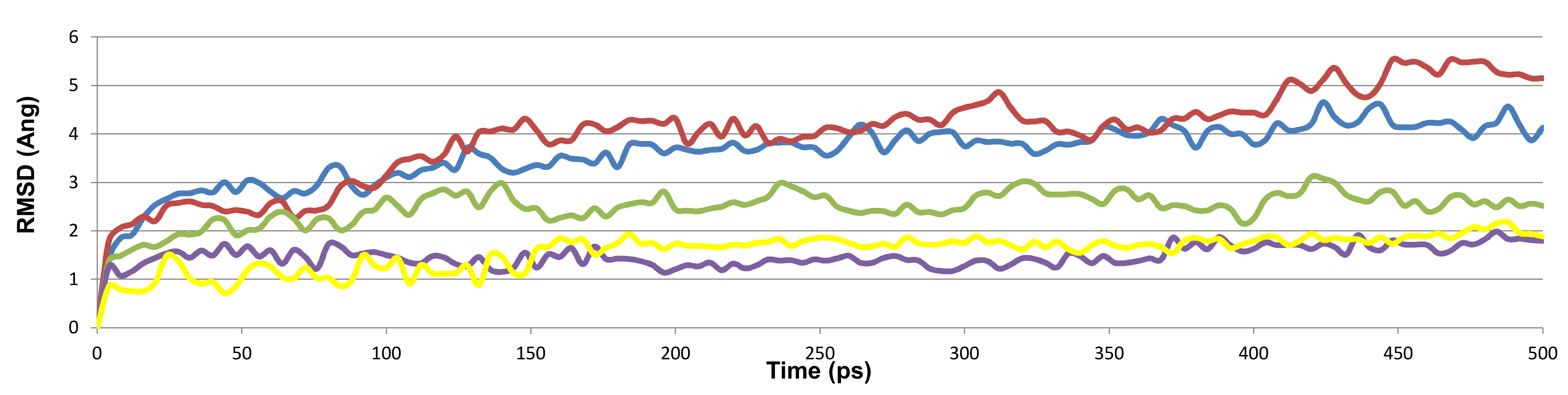

Supplement: Figure S10 — Time dependence of the RMSD of ligand 1 and 2 on the complex (cyan and yellow, respectively) and A and G-quartet heavy atoms (blue) with ligand atoms (1 and 2 are shown in red and green, respectively) at 500 K. (TIF) [file pone.0057701.s010.tif]
