# Supplementary material for: Structure and Stability of Human Telomeric G-Quadruplex with Preclinical 9-Amino Acridines
Source: PLoS One. 2013 Mar 15;8(3):e57701. doi: 10.1371/journal.pone.0057701 (PMC3598906; doi:10.1371/journal.pone.0057701)
Supplement: Table S1 — Logarithm of the binding constants calculated using Equispec program. n.d. not determinded due to lack of changes in the fluorescence spectra. (DOCX) [file pone.0057701.s011.docx]

**Structure and stability of human telomeric G-quadruplex with preclinical 9-amino acridines**

**Table S1.** Logarithm of the binding constants calculated using Equispec program. n.d. not determinded due to lack of changes in the fluorescence spectra.

| **DNA** | **1** | **2** |
| --- | --- | --- |
| Htel | 4.9 ± 0.1 | 4.4 ± 0.1 |
| ds6 | n.d. | n.d. |
