# Supplementary material for: Structure and Stability of Human Telomeric G-Quadruplex with Preclinical 9-Amino Acridines
Source: PLoS One. 2013 Mar 15;8(3):e57701. doi: 10.1371/journal.pone.0057701 (PMC3598906; doi:10.1371/journal.pone.0057701)
Supplement: Table S2 — Selected 1H chemical shift assignments for ds24 (δ) in the presence of 1 a. (DOCX) [file pone.0057701.s012.docx]

**Table S2.** Selected ^1^H chemical shift assignments for ds24 (δ) in the presence of **1***^a^*

| **ds24/1** | **H2/H8/H6** | **H1'** | **NH/H5/CH_3_** |
| --- | --- | --- | --- |
| **A1** | 7.74, | 5.60 | - |
| **A2** | 7.89, | 5.52 | - |
| **G3** | 7.50 | 5.16 | - |
| **A4** | 7.88, | 5.79 | - |
| **A5** | 7.94, | 5.97 | - |
| **T6** | 6.96 | 5.96 | 1.07 |
| **T7** | 7.20 | 5.73 | 1.33 |
| **C8** | 7.38 | 5.82 | 5.39 |
| **T9** | 7.31 | 5.93 | 1.54 |
| **T10** | 7.34 | 6.05 | 1.54 |
| **G3C8** |  |  | 12.13 |
| **A4T7** |  |  | 13.58 |
| **A5T6** |  |  | 13.42 |

^a^ Measured in ppm at 15°C, *R* = [drug]/[DNA] = 4.0
